# Key Gene and Functional Pathways Identified in Unexplained Recurrent Spontaneous Abortion Using Targeted RNA Sequencing and Clinical Analysis

**DOI:** 10.3389/fimmu.2021.717832

**Published:** 2021-08-05

**Authors:** Heng Gu, Longyu Li, Mengxuan Du, Hang Xu, Mengge Gao, Xiaohua Liu, Xiangcai Wei, Xingming Zhong

**Affiliations:** ^1^Key Laboratory of Male Reproduction and Genetics of National Health Council, Family Planning Research Institute of Guangdong Province, Guangzhou, China; ^2^Dongguan Institute of Reproduction and Genetics, Dongguan Maternal and Children Health Hospital, Dongguan, China; ^3^Department of Public Health and Preventive Medicine, Jinan University, Guangzhou, China; ^4^Department of Reproductive Immunity, Guangdong Women and Children Hospital, Guangzhou Medical University, Guangzhou, China

**Keywords:** differentially expressed genes, recurrent spontaneous abortion, ribonucleic acid sequencing, interferon gamma, Th1/Th2 imbalance

## Abstract

Identifying the mechanisms underlying unexplained recurrent spontaneous abortion (URSA) can help develop effective treatments. This study provides novel insights into the biological characteristics and related pathways of differentially expressed genes (DEGs) in URSA. Nineteen patients with URSA and three healthy fertile women with regular menstruation (control group) were recruited. RNA was extracted from the two groups to determine the differential expression of immunoregulatory gene sequences. Gene ontology (GO) and Kyoto Encyclopaedia of Genes and Genomes (KEGG) enrichment analyses were used to identify the biological functions and pathways of the identified DEGs. A protein-protein interaction (PPI) network was constructed using the STRING database. Furthermore, qRT-PCR and ELISA were performed to validate the differential expression of the hub genes. We also explored the regulatory mechanism of Th1/Th2 imbalance. A total of 99 DEGs were identified, comprising 94 upregulated and five downregulated genes. Through GO analysis, nine immune cell function-related clusters were selected, and genes with significant differential expression were primarily enriched in eight immune regulatory functions related to the KEGG signalling pathway. Subsequently, five hub genes (*TLR2, CXCL8, IFNG, IL2RA,* and *ITGAX*) were identified using Cytoscape software; qRT-PCR confirmed the differential expression among the hub genes, whereas ELISA revealed a significant difference in extracellular IFN-γ and IL-8 levels. The levels of Th1 (IFN-γ) and the Th1/Th2 ratio were higher in the peripheral blood of URSA patients than in control group patients. These findings suggest that the occurrence of URSA may be associated with the abnormal expression of some specific immunoregulatory genes involved in T-cell activation and differentiation. Among the identified DEGs, *IFNG* may play a key role in regulating maternal immune response. Although further validation is required, our data provide an important theoretical basis for elucidating the pathogenesis of recurrent spontaneous abortion.

## Introduction

Recurrent spontaneous abortion (RSA), one of the most common complications of pregnancy, refers to two or more consecutive spontaneous abortions with the same partner (1). It occurs in up to 5% of women of reproductive age (2). RSA has a complex aetiology which includes genetic factors, endocrine disorders, anatomical and structural abnormalities, thyroid dysfunction, infectious diseases, coagulation mechanism disorders, and immune factors (3–7). Embryonic chromosomal abnormalities are a major cause of spontaneous abortion during the first trimester; multiple studies have shown that at least 50% of first trimester miscarriages are associated with embryonic chromosomal abnormalities (8–10). However, there remain 40% to 50% of patients with unexplainable miscarriage, referred to as unexplained recurrent spontaneous abortion (URSA) (11). Previous studies have suggested that RSA may be associated with uncharacteristic chromosomal microstructure, gene expression, and noncoding RNA expression variation (12–14).

Mammalian fertilisation as well as embryo implantation and developmental processes are regulated by hundreds of genes (15–17). Several genes involved in the immune response (*IFNG, IL10, KIR2DS2, KIR2DS3, KIR2DS4, MBL*, and *TNF*), coagulation (*F2, F5, PAI-1*, and *PROZ*), metabolism (*GSTT1* and *MTHFR*), and angiogenesis (*NOS3* and *VEGFA*) have been shown to be pathogenetically associated with RSA (18). Most of these genes are associated with overactive immune and inflammatory responses, hypercoagulability, and disturbed metabolic regulation that may contribute to the pathogenesis of RSA. Previous studies have shown that multiple factors involved in immune regulation play a key role in maintaining balance in immune maternal foetal tolerance (19). Indeed, a balance in the immune system influences successful pregnancies, and cytokines secreted by immune cells play important roles in that balance at different stages of implantation. Therefore, in this study, we investigate the potential factors associated with miscarriage in patients with URSA, such as abnormal expression of certain genes that are associated with immune dysfunction. We applied a targeted RNA sequencing chip to screen differentially expressed genes (DEGs) in the peripheral blood of URSA patients, connected to the clinical characteristics, trace the action pathways of the DEGs, and elucidate the underlying immunological mechanisms of URSA at the gene expression level.

## Materials and Methods

### Subjects

#### URSA Group

In total, 19 patients with URSA attending the Guangdong Family Planning Hospital from March 2020 to September 2020 were enrolled in the study. The inclusion and exclusion criteria for the URSA group were as follows: (1) two or more consecutive pregnancy losses before 12 weeks of gestation; (2) the examination of tissues from aborted embryos suggested a normal karyotype; (3) both biological parents had normal chromosomal karyotypes; (4) no genital tract malformations; (5) no reproductive tract infections; (6) menstrual cycle, basic sex hormones, thyroid function, and fasting blood sugar were normal; (7) autoantibodies (antinuclear antibodies, anti-thyroid autoantibodies, anti-phospholipid antibodies) were negative; (8) routine semen examination of the father was normal, excluding those with anatomical, microbial, viral, hormonal, or genetic disease in both couples.

#### Control Group

Throughout the same period, three women who visited the hospital for routine physical examination were selected as the control group. The inclusion and exclusion criteria for these individuals were: (1) given birth at least once; (2) no history of adverse pregnancy events such as spontaneous abortion, stillbirth, or premature delivery; (3) no pregnancy complications such as gestational diabetes or preeclampsia; (4) normal menstrual cycle; (5) no personal or family history of autoimmune system diseases or metabolic diseases.

In this study, peripheral blood samples (10 ml) were collected in the morning during nonpregnancy and nonmenstrual periods after at least 8 h of fasting from all subjects, and 5 ml blood was added to anticoagulant tubes containing 2% ethylenediaminetetraacetic acid (EDTA), which were subsequently stored at −80°C. Another 5 ml blood was centrifuged at 1,760*g* for 10 to 15 min, and the separated serum was stored in tubes at −80°C.

#### Preparation and Hybridisation of Immunomodulatory Gene Chips

Total RNA was extracted with an QIAamp RNA Blood Mini Kit (Qiagen, Cat. No. 52304) following the manufacturer’s protocol and its quality was first examined using an Agilent Bioanalyzer 2100 (Agilent, USA). The purified RNA was used as a template to synthesise cDNA using oligo (dT) as a primer. The experimental procedure was carried out in accordance with the instructions of the reverse transcription kit (Takara, Cat. No. RR036A). After the determination of nucleic acid concentration by using Qubit 3.0 fluorometer (ABI, USA), the targeted gene region transcriptome RNA expression sequencing hybridisation chip was used for RNA capture and target enrichment to the formation of sequence libraries, sequencing was performed on Ion PGM platform (ABI, USA). Further, 212 target genes (detailed in supplementary table) that were mainly related to biological processes—including proliferation, apoptosis, migration, and differentiation of T and B cells and natural killer cells (NK cells), as well as apoptosis and migration of macrophages—were custom designed on a hybridisation chip by Agilent. The entire process was performed in the Guangzhou Ardent Clinical Laboratory.

### DEG Screening

Raw data were trimmed of adapter and primer sequences in reads hereafter low-quality bases using fastp software (version 0.19.7) (20) to obtain clean reads. Subsequently, aligned the clean reads to the reference human genome hg38 using HISAT software (version 2.1.0) (21). Genes expression was calculated using SringTie software (version 1.3.5) (21) based on the alignment. The expression levels of different samples were then normalised using DESeq2 (version 1.22.2) (22) and subjected to differential analysis; DEGs were screened using the “limma” package (23), and a volcano map of DEGs was drawn using the “ggplot2” package (24) to show the differential expression of DEGs; genes with a p-value ≤ 0.05, and an absolute value of log2 fold-change greater than 1 were screened as DEGs.

### GO and KEGG Enrichment Analysis

To explain the biological process and molecular mechanism of DEGs, the biological functions and pathway enrichment were analysed using Gene Ontology (GO) and KEGG enrichment analyses. The enrichment and a series of functional annotation were performed on an online bioinformatics resource website with the name of the Database for Annotation, Visualization, and Integrated Discovery (DAVID, http://david.ncifcrf.gov; version 6.8). The functions of genes and proteins related to three main categories, biological process (BP), cytological component (CC), and molecular function (MF), are defined as described among a standard semantic vocabulary as a GO database, whereas biological interpretation of genome sequences and other high throughput data were contained in the KEGG integrated database (25). Cluster Profiler (version 3.10.1) (26) was used to perform GO and KEGG enrichment analyses and unsupervised hierarchical clustering of DEGs. Heat maps were used to show the expression patterns of DEGs between the case and control groups. A p-value < 0.05 was considered statistically significant in the enrichment in characteristic biological functions in GO annotation and among specific potential pathways in KEGG annotation.

### PPI Network Analysis

Protein-Protein interaction (PPI) regulates several cellular processes including replication, transcription, translation, splicing, secretion, cell cycle, signal transduction, and intermediate metabolism. In this study, the PPI network of DEGs was constructed using the Search Tool for the Retrieval of Interacting Genes (STRING, http://string-db.org; version 11.0b) (27) with an interaction score ≥ 0.4.

### Hub Gene Selection and Analysis

The top five DEGs were defined as hub genes based on the ranking of nodes’ score calculated with the maximum correlation criteria with the topological analysis method of maximal clique centrality algorithm *via* the CytoHubba plugin of Cytoscape (28, 29).

### Real-Time Quantitative PCR

Total RNA was extracted using a QIAamp RNA Blood Mini Kit (Qiagen, Cat. No. 52304) following the manufacturer’s protocol. The purified RNA was used as a template to synthesise cDNA by using oligo (dT) as a primer. The experimental procedure was carried out in accordance with the instructions of the reverse transcription kit (Takara, Cat. No. RR036A). To validate the expression differences of hub genes between the URSA case and normal control groups, according to the results of the previous transcriptome expression sequencing of targeted genes, qRT-PCR was conducted using TB Green Premix Ex Taq™ (Takara, Cat. No. RR820A) to detect the hub genes in a StepOnePlus^™^ Real-Time PCR System with Tower (ABI, USA). Three assays were carried out for each sample. Data were analysed with the 2^−ΔΔCt^ method using ACTB as the internal control. The primers used in qRT‐PCR are listed in **Table 1**.

**Table 1:** Primers for hub genes validated using reverse transcription polymerase chain reaction.

Genes	Accession No	Primer	Sequence(5′-3′)
IFNG	NM_000619.3	IFNG-F	TCCAAGTGATGGCTGAACTG
		IFNG-R	CTCTTCGACCTCGAAACAGC
ITGAX	NM_000887.5	ITGAX-F	GTGGTGGTGTGATGCTGTTC
		ITGAX-R	ATACTGCAGCCTGGAGGAGA
TLR2	NM_001318787.2	TLR2-F	TGATGCTGCCATTCTCATTC
		TLR2-R	CGCAGCTCTCAGATTTACCC
IL2RA	NM_000417.3	IL2RA-F	ATCAGTGCGTCCAGGGATAC
		IL2RA-R	GACGAGGCAGGAAGTCTCAC
CXCL8	NM_000584.4	CXCL8-F	GTGCAGTTTTGCCAAGGAGT
		CXCL8-R	CTCTGCACCCAGTTTTCCTT

### Enzyme Linked Immunosorbent Assay

All peripheral blood samples were collected with suited EDTA anticoagulant tube, stewing at 37°C for 30 min and centrifuged for 15 min at the 1,760*g* to remove supernatant.

ELISA kits were used to detect the concentrations cytometric factors (IL-8, IFN-γ, IL2Rα and TLR2) *via* double antibody sandwich method. Purified capture antibodies of the cytometric factor were coated on a microtiter plate and formed a solid-phase antibody. The revealed cytometric factors in the plasma were captured using HRP-labelled (horseradish peroxidase-labelled) solid-phase antibody to form the antibody-antigen-enzyme labelling-antibody complex and coloured by adding substrate of TMB (3,3′,5,5′-Tetramethylbenzidine) after thorough washing. TMB was transformed into the colour blue under the catalysis of HRP enzyme, and yellow under the effect of acid.

The destination intensity of the colour is positively correlated with the concentration of the cytometric factors in the plasma. We detected the absorbance (OD value) using enzyme-labelled instrument of Infinite M Plex microplate reader (Tecan, Männedorf, Switzerland) at wavelength of 450 nm to calculate the concentration through standard curve.

ELISA kits (MLBio, China) used in this study provided the R value of the correlation coefficient between the linear regression of the sample and the expected concentration above 0.95, whereas the intra-assay and inter-assay coefficient of the variation is less than 10% and 15% respectively.

### Retrospective Cytokine Data Analysis

Th1 (IFN-γ), Th2 (IL-4), and Th1/Th2 ratio data were retrospectively analysed from 227 URSA patients and 18 healthy fertile women attending the Guangdong Family Planning Hospital from January 2014 to December 2018, using the same inclusion and exclusion criteria listed in 2.1.

The levels of Th1 (IFN-γ),Th2 (IL-4), and Th1/Th2 cytokines were detected in fresh peripheral blood within 8 h using BD FACSCalibur flow cytometry (BD Biosciences, San Jose, CA, USA). Briefly, 150 μl of fresh peripheral blood sample within 8 h was placed in a flow tube and 150 μl of RPMI1640 (without calf serum FBS), 10 μl of 1 μg/ml PMA, 10 μl of 50 μg/ml ionomycin, and 10 μl of 0.5 mg/ml BFA were added to the working solution. The plates were incubated at 37°C in 5% CO_2_ for 3 to 6 h. After addition of 10.0 μl CD3percp and 2 μl CD8apc, the mixture was incubated for 15 min in the dark at room temperature, followed by membrane disruption and the addition of IFN-γ/IL-4 antibody. After incubation in the dark at room temperature for 20 min, and the supernatant was discarded after centrifugation and used for detection.

### Statistical Analysis

SPSS 26 software was used for data analysis. The data of general characteristics and laboratory measurement results were tested for normality, and data with a normal distribution were tested for homogeneity of variance. Comparison of data between two groups was conducted using Student’s *t*-test, and those that did not conform to normal distribution were tested with the rank test. Statistical significance was set at *p* < 0.05. GraphPad Prism6 software was used to generate histograms depicting the Th1 (IFN-γ), Th2(IL-4), and Th1/Th2 ratios.

## Results

### General Characteristics and Laboratory Measurements

A comparison of the basic clinical characteristics of the two groups is shown in **Table 2**. The ages of the subjects ranged from 27 to 35 years, with no significant differences in the mean age, weight, and various hormonal contrasts between the two groups (*p* > 0.05).

**Table 2:** Comparison of basic clinical characteristics between case and control group.

	Control group (n = 3)	Case group (n = 19)	*p*
age (years old)	31.67 ± 2.08	30.26 ± 2.60	0.387
Height(m)	1.59 ± 0.02	1.59 ± 0.04	0.787
Weight (kg)	60.33 ± 6.81	56.84 ± 9.11	0.535
BMI (kg/m^2^)	23.84 ± 2.36	22.52 ± 3.27	0.512
FSH (mIU/ml)	6.69 ± 0.45	6.90 ± 1.12	0.748
LH (mIU/ml)	6.25 ± 2.34	8.60 ± 5.55	0.857
LH/FSH	0.92 ± 0.29	1.30 ± 0.95	0.857
E2(pmmol/L)	170.73 ± 29.28	207.14 ± 62.79	0.343
PRL (mIU/L)	920.00 ± 573.15	430.11 ± 263.26	0.108
T(nmol/L)	0.81 ± 0.10	0.94 ± 0.74	0.787

Data are shown as mean± SD; BMI, body mass index; FSH, follicle-stimulating hormone; LH, luteinizing hormone; E2, estradiol; PRL, prolactin; T, testosterone; p value < 0.05 was considered as significant.

### Identification of DEGs

**Figure 1:**
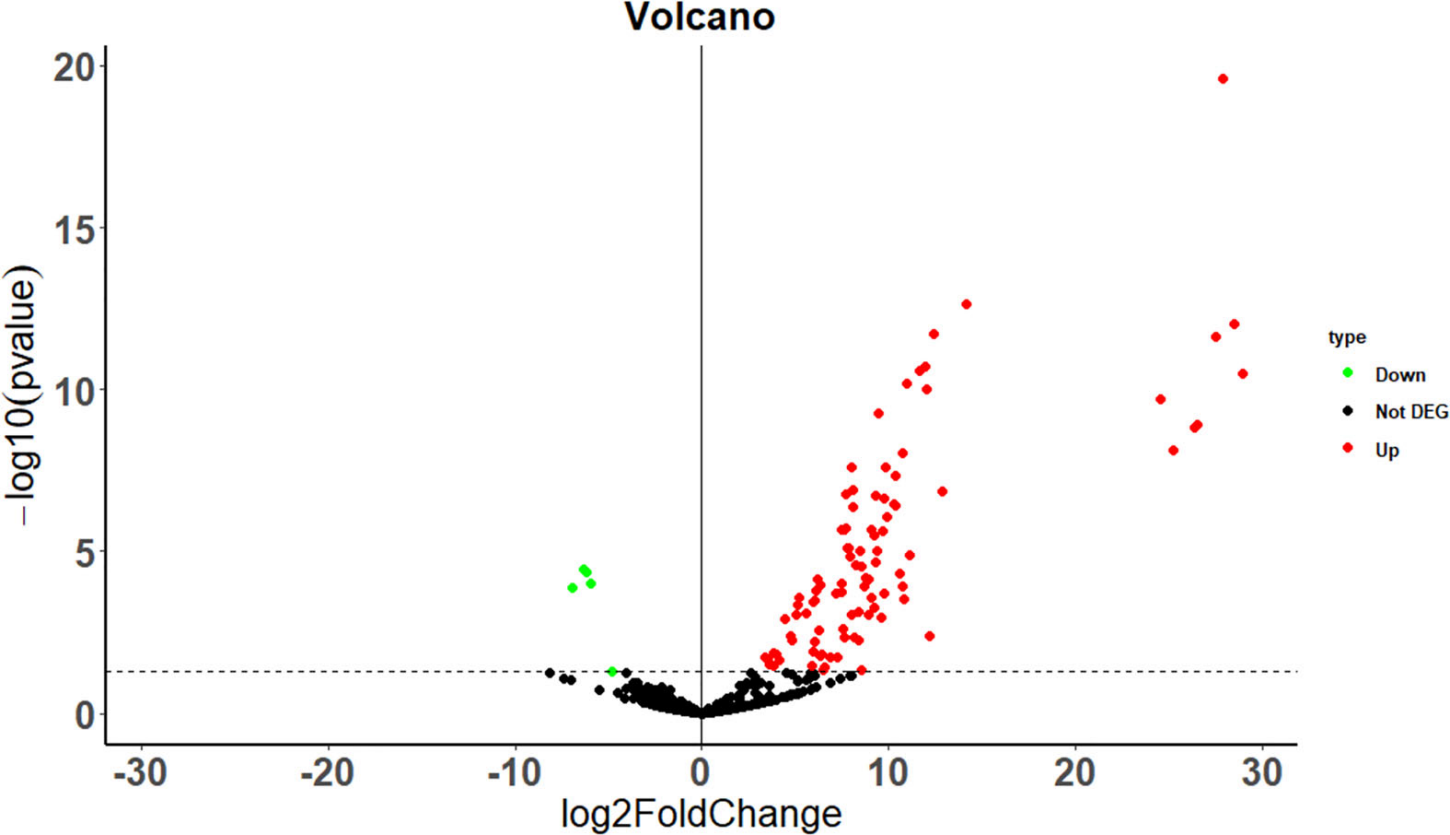
Differential gene volcano map between the case and the control group. The dotted line indicates *p* = 0.05.

The differential expression of immune regulated genes was selected by calculating the *p* value and fold-change value of the chip probes between the case and control groups. A total of 99 DEGs, including 94 upregulated (log2 fold-change > 1, *p* < 0.05), and five downregulated (log2 fold-change < -1, *p*-value < 0.05) DEGs were filtered, and the results are illustrated in **Figure 1** as volcano plots.

### GO and KEGG Enrichment Analyses of DEGs

GO analysis indicated that the 99 DEGs were mainly enriched in processes involved in the T cell activation and proliferation and regulation of leukocyte differentiation (**Table 3**). The results of KEGG pathway enrichment analysis demonstrated that the DEGs were significantly enriched in RSA and immune regulated-associated signalling pathways of NK cell-mediated cytotoxicity and cytokine-cytokine receptor interaction (**Table 4**).

**Table 3:** Immune cell function GO clustering of significantly differentially expressed gene.

ID	Function	Type	Count	*p* value
GO:0042110	T cell activation	BP	19	4.60E-14
GO:1903706	regulation of hemopoiesis	BP	15	2.78E-10
GO:0051249	regulation of lymphocyte activation	BP	15	1.48E-09
GO:0030098	lymphocyte differentiation	BP	14	2.27E-10
GO:0030217	T cell differentiation	BP	13	2.27E-11
GO:1902105	regulation of leukocyte differentiation	BP	13	1.07E-10
GO:0050863	regulation of T cell activation	BP	13	6.07E-10
GO:0046651	lymphocyte proliferation	BP	12	1.42E-09
GO:0032943	mononuclear cell proliferation	BP	12	1.55E-09

p value < 0.05 was considered as significant.

**Table 4:** KEGG enrichment analysis results of significant gene DEGs between the case group and the control group.

ID	Description	Count	*p* value
hsa04650	Natural killer cell mediated cytotoxicity	9	1.34E-06
hsa04145	Phagosome	9	4.20E-06
hsa04060	Cytokine-cytokine receptor interaction	9	0.000276395
hsa05140	Leishmaniasis	8	1.13E-07
hsa05145	Toxoplasmosis	8	1.13E-05
hsa05200	Pathways in cancer	8	0.00504406
hsa04612	Antigen processing and presentation	7	2.98E-06
hsa05323	Rheumatoid arthritis	7	9.04E-06

p value < 0.05 was considered as significant.

### PPI Network and Hub Gene Analysis

**Figure 2:**
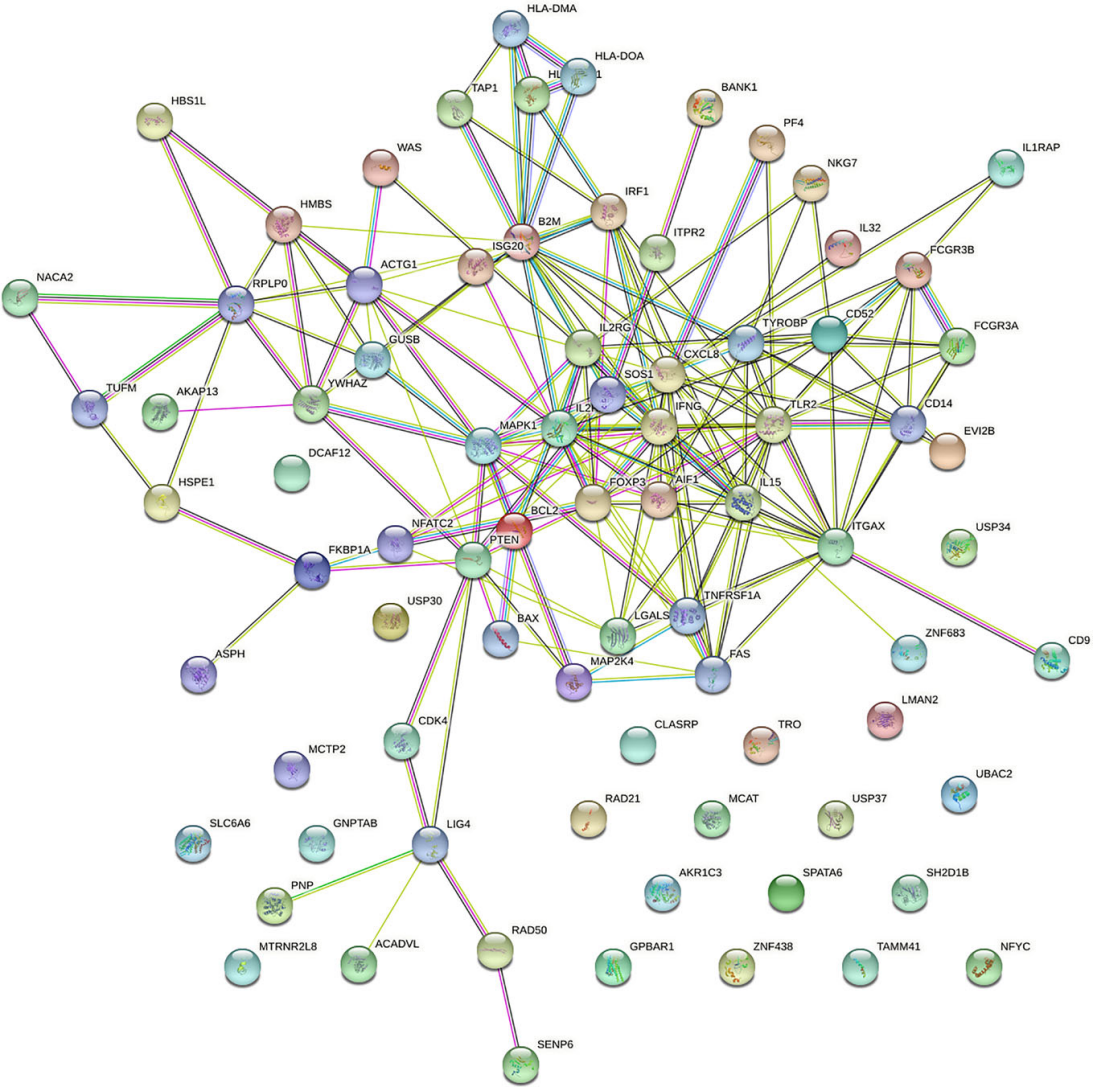
Protein–protein interaction (PPI) network of the dysregulated genes.

**Figure 3:**
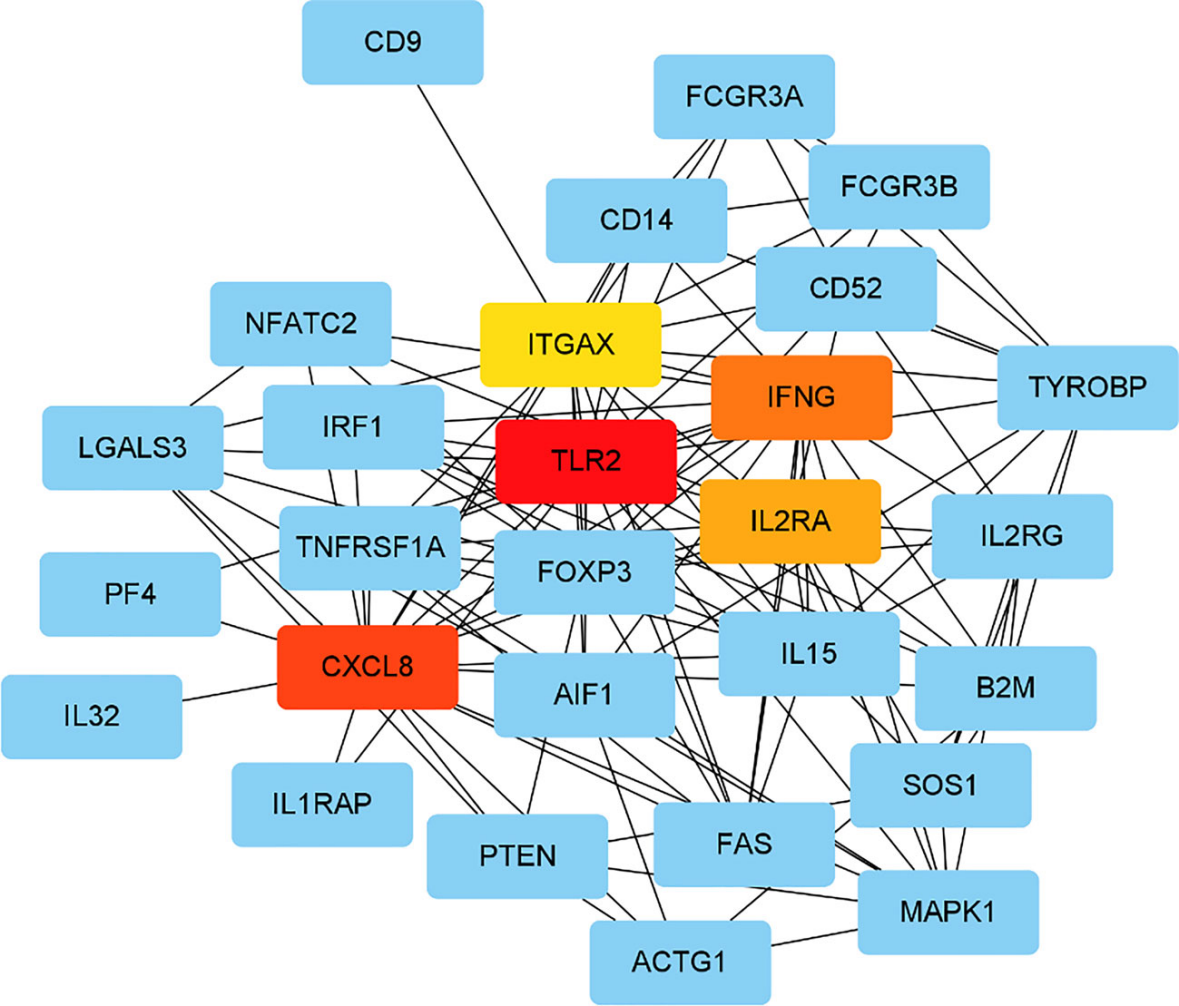
Identified the hub gene by Cytohubba. In the network, TLR2,CXCL8, IFNG, IL2RA, and ITGAX were calculated as the top 5 hub genes.

PPI network was analysed using the STRING database; 81 nodes and 205 edges with an average node degree of 5.06 were established (**Figure 2**). Five hub genes (*TLR2, CXCL8, IFNG, IL2RA* and *ITGAX*) out of the 99 included were identified using CytoHubba according to the nodes’ score ranking using the maximum correlation criteria and the topological analysis method of maximal clique centrality algorithm *via* the CytoHubba plugin of Cytoscape. The PPI network was analysed using the STRING database; the abridged version with the hub gene indicated is shown in **Figure 3**.

### qRT-PCR Validation

**Figure 4:**
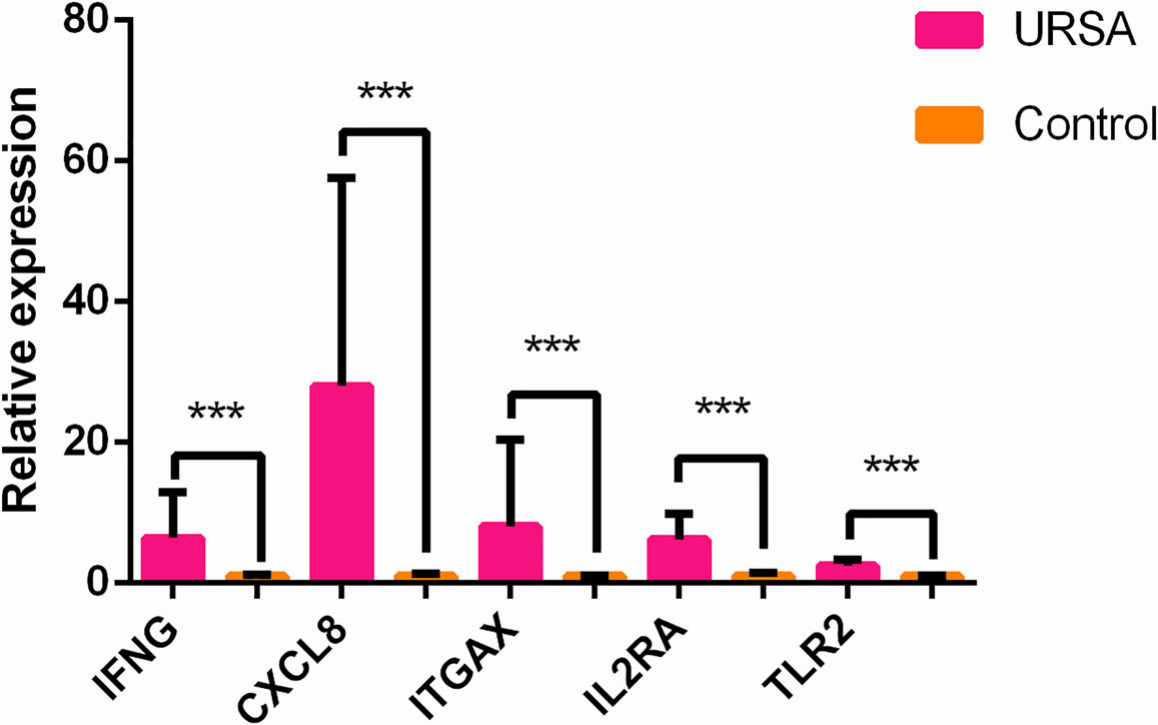
Different expression of the top 5 hub genes in cases and controls verified using qRT-PCR. ***p < 0.05.

Based on the results of the previous targeted transcriptome expression sequencing, RT-PCR was used to validate the high/low expression of hub genes and housekeeping genes differentially expressed in the peripheral blood samples of cases *versus* controls. Differences in *TLR2, CXCL8, IFNG, IL2RA*, and *ITGAX* genes between the URSA and control groups were statistically significant (p < 0.05), which were consistent with the RNA sequencing results (**Figure 4**).

### ELISA Validation

ELISA validation experiment was performed on revealing cytokines and receptors including IFN-γ, IL-2Rα, IL-8, and TLR2 among the results of hub genes analysis to validate the revealing protein concentration in serum between control and case groups. The analytical measurement range of each ELISA assay was as follows: 6.25-200 pg/ml for IL-8 (cat. no. ml077386-2, MLBio, China); 25-800 pg/ml for IFN-γ (Cat. No. ml077386-2, MLBio, China); n25-800 pg/ml for IL2Rα (cat. no. ml367803-2, MLBio, China); 0.75-24 ng/ml for TLR2 (cat. no. ml057760-2, MLBio, China).

**Figure 5:**
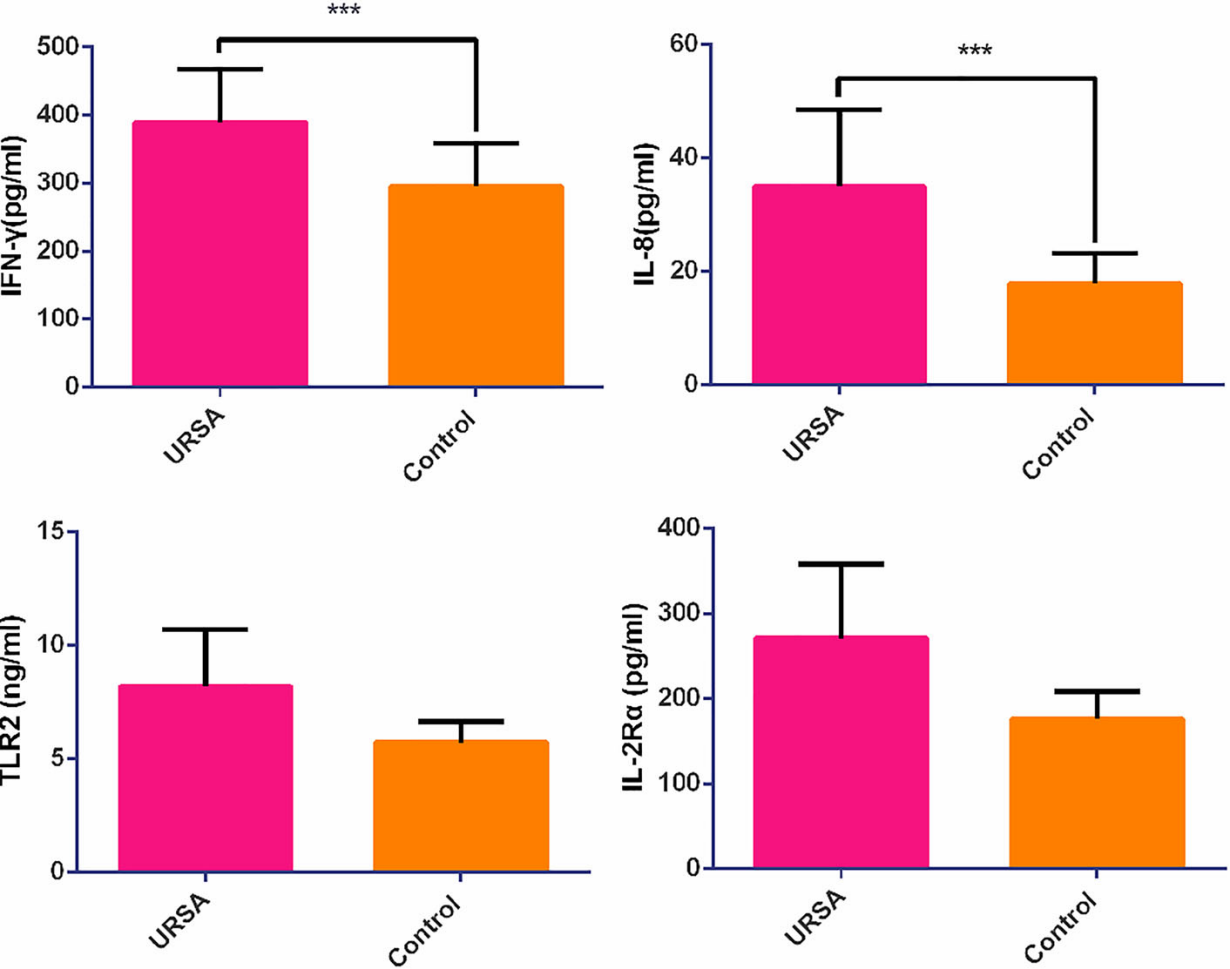
The relative expression of cytokine and chemokine receptors in case and control groups. ***p < 0.05.

As shown in **Figure 5**, IFN-γ and IL-8 were significantly different between the control and case groups (295.36 ± 62.69 *versus* 389.05 ± 77.82 pg/ml, p = 0.040 in IFN-γ and 17.95 ± 5.29 *versus* 34.97 ± 13.59 pg/ml, p = 0.005 in IL-8, respectively), whereas IL-2Rα and TLR2 were not significantly different (176.56 ± 31.49 *versus* 270.93 ± 87.17 pg/ml, *p* = 0.086 in IL-2Rα, and 5.74 ± 0.91 *versus* 8.20 ± 2.50 ng/ml, *p* = 0.113 in TLR2, respectively) using the rank test.

### Retrospective Analysis of Cytokines

**Figure 6:**
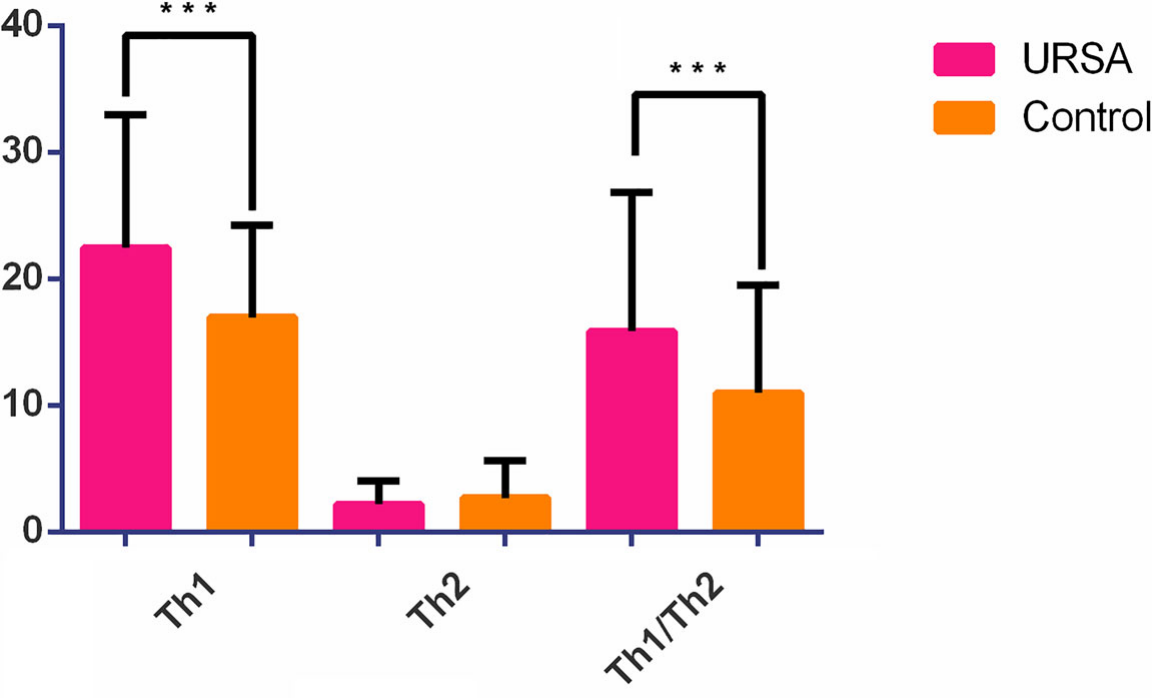
Histogram of Th1 (IFN-γ), Th2 (IL-4), and Th1/Th2 ratio. ***p < 0.05.

Th1 (IFN-γ), Th2(IL-4), and Th1/Th2 cell ratio data from 227 URSA patients who attended the Guangdong Family Planning Hospital from January 2014 to December 2018 and 18 healthy female controls showed that the Th1 (IFN-γ) and Th1/Th2 ratios were significantly higher in the case group (*p* < 0.05), whereas the Th2 (IL-4) ratios were not significantly different (**Table 5** and **Figure 6**).

**Table 5:** Comparison of Th1, Th2, Th1/Th2 between the case group and the control group.

Cohort	Control (n = 18)				URSA (n = 227)				*p* value
variable	Mean	Median	Range	SD	Mean	Median	Range	SD	
Th1*	16.94	15.80	7.60-34.39	7.26	22.44	21.37	0.16–61.42	10.50	0.031
Th2	2.68	1.62	0.39-13.40	2.95	2.19	1.52	0.10–10.45	1.85	0.460
Th1/Th2*	11.00	9.06	0.84-31.87	8.51	15.85	12.74	1.60–55.16	11.01	0.049

*p < 0.05.

## Discussion

Among all RSA causes, the well-defined causes include genetic factors, endocrine factors, thrombosis, immune factors, and genital malformations, whereas at least 50% of RSA remain unexplained and categorised as URSA. It has been suggested that aberrant gene expression is a major cause of URSA and an important indicator of pregnancy disorders (30, 31). The regulation of genes in the embryo includes the self-regulation of genes critical for embryonic development and the biparental genetic influence on embryonic development (32). During early pregnancy, the expression of maternal genes plays a crucial role in embryonic development. The involvement of genetic clusters in the occurrence of miscarriage warrants further investigations to improve our understanding of this complicated condition. It has been shown that immune factor dysfunction plays an important role in the pathogenesis of URSA (33). Utilising gene variation and expression to determine the differences in the expression of immune regulatory genes in the pathogenesis of URSA may help to accurately identify the potential risk and associated factors of URSA to ameliorate pregnancy outcomes.

In this study, 99 DEGs between the URSA and control groups were significantly enriched in T-cell activation and proliferation, and regulation of leukocyte differentiation. Meanwhile, most genes in the relevant clusters were significantly upregulated in differentially expression states, suggesting that significant upregulation of genes in relevant immunoregulatory clusters may induce immune dysfunction in URSA patients. According to PPI analysis, *TLR2, CXCL8, IFNG, IL2RA*, and *ITGAX* were among the top five immunoregulatory hub genes of DEGs. KEGG pathway analysis revealed that the main DEGs in URSA patients, compared with normal pregnant women, were enriched in several signalling pathways associated with immune regulatory functions, including natural killer cell-mediated cytotoxicity, cytokine receptor interaction, and other signalling pathways. NK cells are present in the endometrium and synergise with T lymphocytes to generate immune tolerance at the maternal foetal interface, which is important for a successful pregnancy (34, 35). Abnormal gene expression of cytokines and cytokine receptor signalling pathway suggest immune cell dysregulation in women with recurrent pregnancy loss.

The protein encoded by *TLR2* is a member of the *TLR* family, which plays an important role in pathogen recognition and innate immune activation and is widely expressed during the maternal foetal interface, including immune cells, trophoblasts, and decidual cells. Multiple studies have shown that TLRs play a crucial role in the pathogenesis of autoimmune diseases, since *TLR* stimulation has been reported to induce foetal resorption during early pregnancy loss (36, 37). *TLR2*, an initiation factor of the TLR signalling pathway, can increase the release of IL-8 through signalling factors such as MyD88 and is critical in recognising microbial infections and mediating innate immune responses (38).

IL-8, encoded by the *CXCL8* gene, is an important mediator in the acute immune response, stimulation of chemoattraction, and promotion of angiogenesis, that may play an important role in URSA by regulating the maternal immune response (39). IL8 recruits neutrophils to the surrounding tissues thereby promoting the spread of acute inflammation and influences the levels of IL6 and IL1 expressed in the foetal membranes of women with chorionicity. Furthermore, IL-8 may control the chemotaxis and migration of immune cells at the site of inflammation and plays a significant role in embryo implantation and the establishment of pregnancy through a network cooperating with other cytokines (40). Antagonising *CXCL8* and its receptor could reduce the inflammatory response and improve the symptoms of inflammation-related diseases (41). However, the role of IL-8 and other cytokines in pregnancy and their prognostic value for pregnancy outcome remains to be fully elucidated.

The *IFNG* gene is located on 12q15 and encodes IFN-γ, which is a Th1 proinflammatory cytokine with multifaceted regulatory effects on immune responses in the body (42). Nakagawa et al. showed that Th1 and Th2 cells play important roles in the immune response, especially in immune rejection and tolerance, and the imbalance of Th1/Th2 ratio is not conducive to maintaining normal pregnancy (43). Tangri et al. (44) demonstrated that Th1 type cytokines, such as IFN-γ and TNF-α, are highly expressed in the placentas of pregnant mice with propensity to miscarriage, but not in normal pregnant mice. The immune response is biased toward a Th2 type during normal pregnancy, which protects the embryo from rejection by the maternal immune system, favouring the implantation of fertilised eggs and the development of the foetus (45). If a Th1/Th2 imbalance occurs during pregnancy, a Th1 type bias with increased secretion of Th1 type cytokines and enhanced cellular immunity may damage the placental trophoblasts and foetus, thereby inducing URSA (46). Animal studies have shown that the key genes in the regulation of maternal immune responses and the maintenance of normal pregnancy is important, since *IFNG* can affect the immune response by regulating the expression of several immune-related genes, including *CIITA, KYNU, IDO1, WARS*, and *MHC* (47). Previous studies have reported the imbalance of Th1/Th2 in URSA females, besides, IFN-γ, an indicator of Th1 bias, encoding genes have been previously screened in animal transcriptome studies. Conversely, in this study, we considered combining the imbalance of Th1/Th2 in URSA and the key genes in the regulation of maternal immune responses identified in previous animal studies to illustrate the significance of the potential indicators in the peripheral blood among the Th1/Th2 differentiation and other signalling pathways.

**Figure 7:**
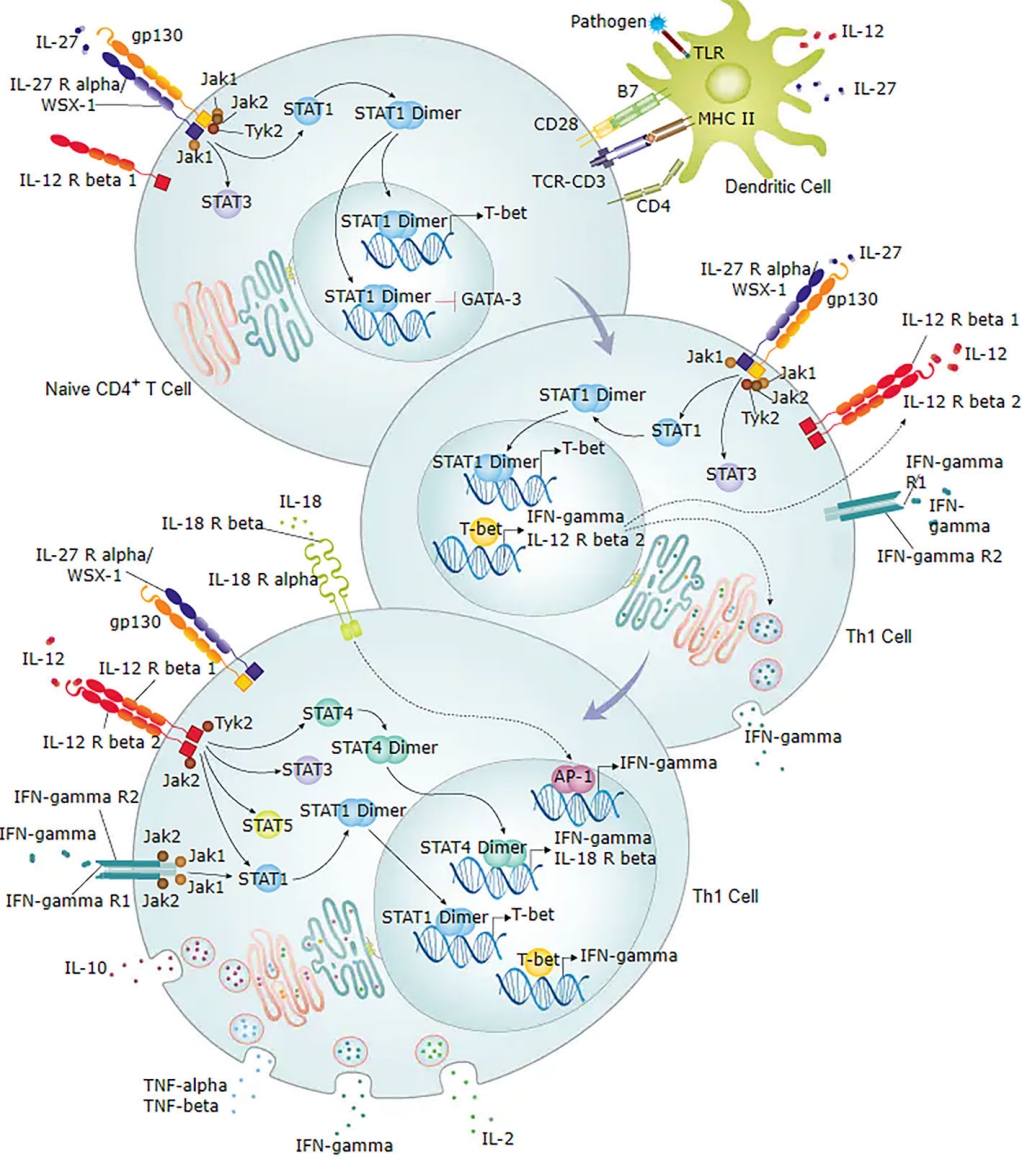
Diagram of IFN-γ involved in Th1/Th2 cell differentiation signalling pathway. Figure obtained from https://www.rndsystems.com/pathways.

In this study, *TLR2, CXCL8, IFNG, IL2RA*, and *ITGAX* genes were upregulated. To further verify the results of the RNA expression, we confirmed the high/low expression of the above genes using qRT-PCT which was consistent with the RNA sequencing results. We also detect these gene-regulated cytokines using ELISA. The levels of IFN-γ and IL-8 were significantly higher in the URSA group than in control. The levels of IL-2R and IL2Rα in the URSA group were higher than in control, but not significantly. Using flow cytometry, we showed that IFN-γ was significantly increased in the peripheral blood of URSA patients. Our results also showed that the high expression of IFNG can promote the secretion of IFN-γ both inside and outside of the cell. IFN-γ, which is an important Th1 cytokine and an initiator of Th17 cell differentiation and the Th1/Th2 cell differentiation signalling pathway, regulates T cell activation through IFNG/STAT1/T-bet signalling as shown in **Figure 7** (48). Meanwhile, T-bet factors within the nuclear envelope act through RUNX1-RORγt, which can inhibit the RUNX1 factor, a positive regulator of Th17 cell differentiation (49, 50), by blocking Th17 cell differentiation and resulting in a Th17/Treg imbalance. Thus, the *IFNG* gene may cause maternal foetal Th1/Th2/Th17/Treg imbalance by regulating the release of IFN-γ, thereby increasing the risk of miscarriage. To further confirm the contribution of the *IFNG* gene to the release of IFN-γ and modulation of the Th1/Th2 balance, we investigated the expression of Th1 (IFN-γ), Th2 (IL-4), and Th1/Th2 ratio in the peripheral blood of 227 URSA patients and 18 normal controls. The results show that the Th1 (IFN-γ) and Th1/Th2 ratios were significantly higher in the URSA group, which suggests that the main cause of the Th1/Th2 imbalance is an increase in Th1 (IFN-γ), possibly associated with the regulation of the *IFNG* gene. Our results suggest that IFNG may play an important role in regulating the maternal immune function.

Qiu et al. have reported that the imbalance of GATA-3/T-bet transcription factors may interfere with type 1 helper T cell (Th1)/type 2 helper T cell (Th2) differentiation at the foetal-maternal interface and possibly cause URSA *via* situ hybridisation (51). Furthermore, Wu et al. observed a Th1/Th2 imbalance and an increase in sTim-3 and Galectin-9 expression in the patients with URSA, which may be involved in the regulation of immunity during pregnancy (52). Recently, Chen et al. screened out *ATP6V1G3*, a key gene in RSA patients, *via* RNA-seq and qRT-PCR validation (25). Therefore, we have performed target region RNA-seq to analyse the expression pattern of the specific immunoregulatory related genes in the peripheral blood of URSA patients, and verified the results of RNA-seq by qRT-PCR. Based on the output of analysis, we summarised the clinical features of immunological disorders in URSA patients for comparison. The data from analysis of bioinformatics and clinical immunology showed that the CXCL8 and IFNG gene we screened out have been confirmed to play an important role in immune imbalance, supporting the reliability of our research. Furthermore, we used ELISA to evaluate the cytokines and their associated receptors from the results of the hub genes analysis to confirm the serum protein concentration between the control and case groups, which provided further evidence of indicators and sensors present in the peripheral blood among the Th1/Th2 differentiation and other signalling pathways. Our results identified a significant indicator, i.e., IFN-γ and IL-8, and additional indicators that are not significant, i.e., IL-2Rα and TLR2 as a reference for future research.

## Conclusions

In this study, several immune-related genes involved in T cell activation and proliferation and cytokine-cytokine receptor interaction were screened from URSA patients. However, gene expression was controlled by multiple factors, such as RNA splicing, regulation of transcription factors, ethylation of the promoter region, and histone modification. Therefore, determining the underlying mechanism of DEGs may contribute to the development of therapeutic strategies against URSA. Another possible limitation of our study is the relatively small sample size, and hence the conclusions cannot be generalised.

In summary, we explored the pathogenesis of URSA at the genetic level and screened the DEGs enriched in immune-related gene clusters while mapping specific immune-associated signalling pathways. The key genes and functional pathways identified in this study provide new insights into the molecular mechanisms involved in URSA pathogenesis and provide potential diagnostic and therapeutic targets. *In vivo* and *in vitro* validation of our findings and elucidation of the specific mechanisms of this cluster in URSA are warranted.

## Data Availability Statement

The original contributions presented in the study are included in the article/**Supplementary Material**. Further inquiries can be directed to the corresponding authors.

## Ethics Statement

The studies involving human participants were reviewed and approved by the Ethics Committee of the Guangdong Family Planning Hospital. The patients/participants provided their written informed consent to participate in this study.

## Author Contributions

All authors contributed to the article and approved the submitted version.

## Funding

This work was supported by Guangzhou Municipal Science and Technology Project (Grant number: 202102080062) and Guangdong Medical Research foundation (Grant numbers: B2021304, A2020467).

## Conflict of Interest

The authors declare that the research was conducted in the absence of any commercial or financial relationships that could be construed as a potential conflict of interest.

## Publisher’s Note

All claims expressed in this article are solely those of the authors and do not necessarily represent those of their affiliated organizations, or those of the publisher, the editors and the reviewers. Any product that may be evaluated in this article, or claim that may be made by its manufacturer, is not guaranteed or endorsed by the publisher.
